# Personalized Calibration and Hybrid Feature Fusion for Continuous Blood Pressure Estimation Using PPG and ECG Signals

**DOI:** 10.3390/s26154676

**Published:** 2026-07-23

**Authors:** Zichuan Zhang, Peng Qu, Hong Tang

**Affiliations:** School of Biomedical Engineering, Dalian University of Technology, No. 2 Linggong Road, Ganjingzi District, Dalian 116024, China; zcc2020@mail.dlut.edu.cn (Z.Z.); qupeng@dlut.edu.cn (P.Q.)

**Keywords:** continuous blood pressure estimation, photoplethysmography, electrocardiography, self-supervised learning, hybrid feature fusion, personalized calibration

## Abstract

**Highlights:**

**What are the main findings?**

- A personalized calibration and hybrid feature fusion framework was developed for continuous BP estimation.
- The proposed method integrates handcrafted physiological features, self-supervised waveform representations, and subject-specific prior BP information.

**What are the implications of the main findings?**

- The integration of physiological interpretability and self-supervised learning improves BP estimation under small-sample conditions.
- This framework shows potential for wearable BP monitoring, although further validation is required before clinical deployment.

**Abstract:**

Continuous and noninvasive blood pressure (BP) monitoring is important for hypertension screening and long-term cardiovascular management. Continuous BP estimation using photoplethysmography (PPG) and electrocardiography (ECG) has become a promising alternative to conventional cuff-based measurements. However, handcrafted feature-based methods are sensitive to fiducial point detection, while end-to-end deep models often require large labeled datasets and may ignore subject-specific BP baselines. This study proposes a personalized calibration and hybrid feature fusion framework for continuous BP estimation from ECG and PPG signals. The framework integrates physiologically interpretable handcrafted features, self-supervised waveform representations, and subject-specific prior BP information to predict systolic and diastolic BP. The handcrafted branch extracts pulse transit time, heart rate variability, and PPG morphological descriptors, whereas a masked reconstruction-based self-supervised encoder learns latent waveform embeddings without BP labels. Personalized calibration incorporates base BP from the earliest calibration file through an alpha-weighted strategy, and the fused representation is fed into XGBoost regressors. The framework was evaluated on a private dataset and the BP-UCI dataset, achieving SBP/DBP MAEs of 7.25/3.98 mmHg and 7.85/4.02 mmHg, respectively. Comparative and ablation results indicate improved baseline performance in the current experimental setting, suggesting the methodological feasibility of the proposed framework for small-sample continuous BP estimation. Further validation with larger and more diverse cohorts is still required.

## 1. Introduction

Cardiovascular disease (CVD) remains one of the leading causes of mortality and disease burden worldwide, and hypertension is among the most important modifiable risk factors [1,2,3]. As a key physiological indicator of cardiovascular function, blood pressure (BP) is closely associated with stroke, heart failure, renal impairment, arterial stiffness, and other cardiovascular complications [1,3]. Therefore, continuous, noninvasive, and convenient BP monitoring is important for early hypertension screening, dynamic risk assessment, and long-term cardiovascular health management [2,4]. Conventional cuff-based BP measurement has been widely used in clinical and home settings; however, it provides only intermittent measurements and is therefore insufficient for continuous hemodynamic monitoring. In addition, repeated cuff inflation and deflation may cause discomfort and interfere with daily activities and sleep [4,5,6].

With the rapid development of wearable sensing and mobile health technologies, cuffless BP estimation based on physiological signals has attracted increasing attention for continuous health monitoring [7,8,9,10]. Among different biosignals, photoplethysmography (PPG) and electrocardiography (ECG) have been widely investigated because they are easy to acquire, low-cost, and suitable for integration into wearable devices [4,5,6]. Compared with conventional cuff-based measurements, ECG/PPG-driven BP estimation has potential advantages in continuous monitoring, user comfort, and device miniaturization.

Existing ECG/PPG-based BP estimation methods can be broadly divided into handcrafted feature-based approaches and data-driven deep learning approaches. Handcrafted feature-based methods usually extract explicit physiological features from ECG and PPG signals, such as pulse transit time (PTT), pulse rise time, waveform amplitude, width, area, systolic slope, heart rate, and heart rate variability, and then employ conventional machine learning models for BP regression [11,12,13,14,15]. PPG derivatives, time-frequency descriptors, and demographic information have also been used to enrich BP-related feature representation [16,17,18]. These features are physiologically interpretable and provide a relatively transparent modeling process. However, their performance strongly depends on signal quality and fiducial point detection. Under motion artifacts, low perfusion, or waveform distortion, feature extraction may become unstable. Moreover, manually designed features cannot fully capture the high-dimensional, nonlinear, and multi-scale temporal patterns embedded in raw physiological signals [4,5,14,15].

To improve feature representation capability, deep learning methods have been increasingly introduced into continuous BP estimation [19,20,21,22,23,24,25]. These methods typically use raw PPG or ECG/PPG waveforms as input and learn nonlinear mappings between physiological signals and BP through convolutional neural networks (CNNs), recurrent neural networks (RNNs), long short-term memory networks (LSTMs), Transformer-based models, attention mechanisms, or hybrid architectures [26,27]. Deep models can capture richer local morphological patterns and long-range temporal dependencies and may achieve strong predictive performance in large-scale datasets. For example, Wang et al. developed PulseDB and emphasized that sample size, subject distribution, data cleaning strategies, and evaluation protocols can substantially affect the fairness of performance comparison [28]. Chen et al. further proposed a PPG/ECG-based framework incorporating residual structures, U-Net encoding–decoding, multi-scale feature fusion, and transfer learning [22]. Nevertheless, end-to-end deep learning models usually require large amounts of labeled data and often lack explicit physiological interpretability. In small-sample biomedical scenarios, purely supervised deep models may also suffer from overfitting and limited generalization [4,5,6,19,21].

To combine the interpretability of handcrafted features with the representation capability of deep learning, hybrid modeling strategies have recently attracted increasing attention. These methods usually incorporate morphological features, physiological priors, or physical constraints into deep networks to enhance the representation of hemodynamic information. Yi et al. proposed a personalized and explainable BP estimation method from single-channel PPG by integrating CNN-based representations with explicit morphological features and feature attribution analysis [29]. However, most existing hybrid approaches are developed under relatively large-scale or dataset-specific settings. How to stably exploit deep representations and effectively combine them with handcrafted physiological features under limited sample conditions remains insufficiently explored.

Personalized calibration is another key factor affecting the practical applicability of cuffless BP estimation. Different subjects may present substantial variations in baseline BP, vascular compliance, pulse propagation characteristics, and sensor wearing conditions. As a result, a population-level model often fails to fully compensate for inter-subject distribution shifts [4,5,6,30,31]. Previous studies have emphasized that calibration strategy, validation protocol, and subject-level testing design are central to evaluating cuffless BP measurement devices [32]. Calibration-stage BP information can also be embedded into the feature space or used to correct subject-specific bias [33,34]. In addition, model evaluation should not rely only on average error, but should also consider whether the validation protocol reflects realistic deployment scenarios [35]. Recent reviews further indicate that robust representation learning, calibration, and standardized evaluation remain important challenges for wearable cuffless BP estimation [36,37].

Overall, previous studies have demonstrated the importance of handcrafted features, deep learning, hybrid modeling, and personalized calibration for cuffless BP estimation [38]. However, under small-sample conditions, how to jointly exploit physiologically interpretable features, raw waveform representations, and subject-specific baseline BP information to improve model stability and cross-dataset generalization remains an open problem. To address this issue, this study proposes a personalized calibration and hybrid feature fusion framework for continuous BP estimation using ECG and PPG signals. The proposed method integrates a handcrafted feature branch, a masked reconstruction-based self-supervised waveform representation branch, and subject-specific prior BP information within an XGBoost-based structured regression framework for systolic BP (SBP) and diastolic BP (DBP) estimation.

The main contributions of this study are summarized as follows:

(1) A calibration-aware hybrid feature fusion framework is proposed for ECG/PPG-based continuous BP estimation. The framework combines physiologically interpretable handcrafted features, self-supervised waveform representations, and subject-specific baseline BP information within a structured XGBoost regression model, aiming to balance physiological interpretability and nonlinear representation capability.

(2) A masked reconstruction-based self-supervised learning strategy is introduced to learn waveform-level temporal representations from raw ECG/PPG windows without requiring BP labels. This branch complements fiducial-point-based handcrafted features, such as PTT, heart rate, PPG morphology, and short-term rhythm variability, by capturing latent morphological and temporal patterns in the raw waveforms.

(3) Subject-specific baseline BP information is incorporated as a practical calibration component to reduce inter-subject baseline deviation. The proposed framework is evaluated on both a private dataset and the public BP-UCI dataset, and ablation studies are conducted to analyze the contributions of self-supervised feature fusion and calibration.

The main methodological novelty of this study lies in the hybrid fusion of self-supervised waveform representations and physiologically interpretable handcrafted features for ECG/PPG-based cuffless BP estimation, aiming to combine latent temporal-pattern learning with explicit cardiovascular feature modeling.

## 2. Materials and Methods

### 2.1. Datasets

The private dataset consisted of ECG, PPG, reference blood pressure (BP), and demographic information collected from 45 subjects, including 33 males and 12 females. During data acquisition, each subject first remained seated at rest, and ECG, PPG, and reference BP values were recorded continuously for approximately 2–3 min. After a short rest period, the subject performed approximately 3 min of cycling exercise, followed by another 3 min of ECG, PPG, and BP recording.

Reference BP values were measured using an OMRON-HME7200 upper-arm BP monitor. All experiments were conducted in a temperature-controlled laboratory environment. The data of each subject were stored in an independent folder, resulting in 240 CSV files in total. ECG and PPG signals were synchronously recorded using an integrated ECG–PPG physiological signal acquisition device. ECG signals were acquired through finger-contact electrodes integrated into the device, while PPG signals were recorded from the fingertip using an optical sensor. Both signals were acquired at the original sampling rate of 1000 Hz, which was determined by the acquisition system. This sampling rate provides a temporal resolution of 1 ms, which helps reduce timing quantization error in ECG R-peak and PPG pulse-peak localization. Since PTT-related features are calculated from the time delay between ECG and PPG fiducial points, millisecond-level resolution is beneficial for reliable feature extraction. The demographic and BP characteristics of the participants, including sex, age, height, weight, BMI, and SBP/DBP distributions, are summarized in Table 1. Written informed consent was obtained from all participants before data collection.

To further evaluate the generalization capability of the proposed method, the BP-UCI dataset was used as an external public dataset. BP-UCI is a sub-dataset of MIMIC-II and contains ECG, PPG, and arterial blood pressure (ABP) signals collected from more than 900 adult ICU patients admitted to Beth Israel Deaconess Medical Center, Boston, MA, USA, between 2001 and 2008. The dataset contains approximately 12,000 anonymized preprocessed signal instances and was specifically curated for cuffless BP estimation research. Compared with the private dataset, BP-UCI provides a larger-scale and more heterogeneous external evaluation setting, which helps assess whether the proposed framework can maintain reasonable performance beyond the 45-subject private cohort. The sampling frequency is 125 Hz.

Because BP-UCI contains a wide range of inter-subject BP variability, it is suitable for evaluating model performance under different subject distributions and BP ranges. In this study, signal quality screening was performed, and 1000 ECG/PPG signal samples were finally selected for evaluation. The selection was based on signal quality and the availability of synchronized ECG, PPG, and ABP segments.

The private dataset was designed as a controlled experimental dataset for methodological validation of cuffless BP estimation rather than as a large-scale clinical population dataset. The acquisition protocol required synchronized ECG, PPG, and reference BP measurements under resting and post-exercise recovery conditions, together with signal-quality assessment and subject-wise evaluation. Therefore, although the number of participants in the private dataset was limited to 45, the dataset provides paired physiological signals and reference BP values for evaluating the feasibility of the proposed personalized calibration and hybrid feature fusion framework. To further assess generalization beyond the private cohort, the proposed framework was also evaluated on the public BP-UCI dataset, which provides a larger number of signal instances and a more heterogeneous BP distribution.

### 2.2. Framework Overview

**Figure 1:**
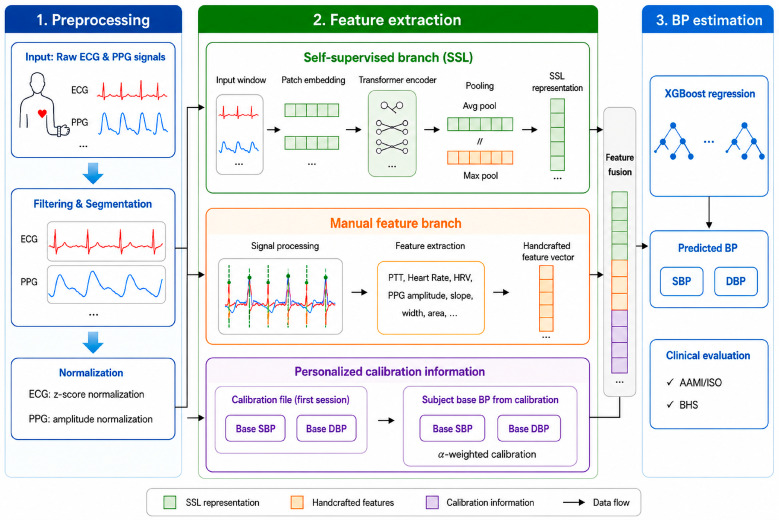
Overview of the proposed calibration-based continuous blood pressure estimation framework. Ellipses indicate omitted intermediate waveform samples, patch tokens, and feature dimensions for schematic simplicity.

We propose a personalized calibration and hybrid feature fusion framework for continuous BP estimation using ECG and PPG signals, as illustrated in Figure 1. The proposed framework takes synchronized ECG and PPG windows as input and consists of four main components: a handcrafted physiological feature branch, a self-supervised raw waveform representation branch, a personalized calibration module, and a structured regression module.

The handcrafted branch extracts physiologically interpretable features from ECG/PPG signals, including pulse transit time (PTT), heart rate variability (HRV), and PPG morphological descriptors. The self-supervised branch learns deep temporal representations from raw ECG/PPG windows using a masked reconstruction task, thereby complementing the information that cannot be explicitly described by handcrafted features. Subject-specific prior BP information is then derived from the calibration file and fused with the handcrafted and self-supervised features. Finally, the fused feature vector is fed into XGBoost regressors to estimate systolic BP (SBP) and diastolic BP (DBP).

The training process includes two stages. First, a self-supervised encoder is pretrained on ECG/PPG windows from the training subjects. Second, the learned waveform representations are concatenated with handcrafted features and subject-specific prior BP information to train separate SBP and DBP regression models.

### 2.3. Signal Preprocessing

Raw ECG and PPG signals were preprocessed before feature extraction. For ECG signals, a second-order Butterworth band-pass filter with a passband of 0.5–35 Hz was applied. For PPG signals, a second-order Butterworth band-pass filter with a passband of 0.5–10 Hz was used to suppress noise, and linear detrending was applied to correct baseline drift.

Because reference BP values were measured intermittently by a cuff-based device, SBP and DBP labels were not sample-wise aligned with the continuous ECG/PPG signals. Linear interpolation was therefore used to construct continuous BP label sequences. ECG signals were standardized using z-score normalization, while PPG signals were normalized to reduce inter-subject amplitude variations.

The preprocessed ECG, PPG, and BP label sequences were segmented into non-overlapping 10 s windows. There is no universally fixed window length for cuffless continuous BP estimation, because the appropriate window length depends on the target task, feature type, and required temporal resolution. In this study, a 10 s window was selected as a trade-off between local prediction stability and temporal continuity for continuous BP monitoring. The purpose of the 10 s window was not to perform long-term spectral analysis or standard long-duration HRV assessment. Instead, it was used to extract local beat-level and waveform features, including PTT, heart rate, PPG morphology, and short-term RR interval descriptors. A 10 s segment generally contains approximately 10–15 cardiac cycles under normal heart rates, which provides multiple ECG–PPG beat pairs for local feature averaging and robust fiducial point detection. Meanwhile, compared with longer windows, a 10 s window can reduce temporal smoothing and prediction delay, which is important for tracking BP changes during post-exercise recovery.

After filtering and segmentation, signal-quality assessment was performed for each 10 s window before feature extraction. The quality-control criteria were designed according to the principles of SQI-based ECG and PPG quality assessment reported in previous studies [39,40]. The purpose of this step was to identify technically unreliable signal segments rather than to remove abnormal but valid physiological responses. Importantly, the quality assessment was based only on signal characteristics and reference-label validity, and was independent of the model prediction errors.

For ECG signals, windows with severe noise, baseline drift, flat-line saturation, or unreliable R-peak detection were regarded as low-quality segments. For PPG signals, windows with missing segments, saturation, distorted pulse morphology, or unreliable systolic peak detection were regarded as low-quality segments. A 10 s window was retained only when reliable ECG R peaks and PPG pulse peaks could be detected, valid ECG–PPG beat pairs could be formed, the derived PTT values were physiologically plausible, and the reference BP labels were within broad validity ranges with SBP > DBP. Windows that failed these criteria were excluded from offline feature extraction because unreliable fiducial points would lead to physiologically meaningless PTT-, HR-, and morphology-related features.

### 2.4. Handcrafted Feature Extraction and SHAP-Based Selection

The handcrafted feature extraction module generated structured physiological descriptors from each valid ECG/PPG window. The initial candidate feature set included time-delay, heart rate, PPG morphological, amplitude-change, and heart rate variability descriptors. These features were designed to characterize pulse propagation delay, cardiac rhythm, waveform morphology, and short-term RR interval variability. Table 2 summarizes the final handcrafted feature groups used for regression modeling.

PTT was defined as the interval between the ECG R peak and the first valid PPG systolic peak after the R peak within the same cardiac cycle. Beat pairs outside the physiological PTT range were excluded. HeartRate was derived from adjacent RR intervals. SysVolChange, SysMaxVolChange, and DiaMaxVolChange represented systolic amplitude variation, maximum systolic upstroke rate, and maximum diastolic downstroke rate, respectively. PWIR described the relative relationship between systolic and diastolic waveform variations. HRV-related descriptors, including SDNN and RMSSD, were calculated from RR interval sequences within each window and were used only as local short-term rhythm variability features rather than as standard long-term HRV indices.

To reduce feature redundancy, SHAP was used for feature importance analysis. In each fold of cross-validation, an initial XGBoost model was trained using only the training set, and SHAP values were calculated on the training samples. The global importance of the j-th feature was defined as

(1) $$\phi_{ij}=\sum_{S\subseteq F\setminus \{j\}}\frac{|S|!(M-|S|-1)!}{M!}\left[f_{i}(S\cup \{j\})-f_{i}(S)\right]$$

(2) $$I_{j}=\frac{1}{N}\sum_{i=1}^{N}\left|\phi_{ij}\right|$$

where F denotes the full feature set, M is the number of features, S is a subset of features excluding the j-th feature, and $f_{i}\left(S\right)$ denotes the model output for the i-th sample when only the feature subset S is available. Where N denotes the number of training samples, $\phi_{ij}$ is the SHAP value of the j-th feature for the i-th sample, and $I_{j}$ is the corresponding global importance score.

**Figure 2:**
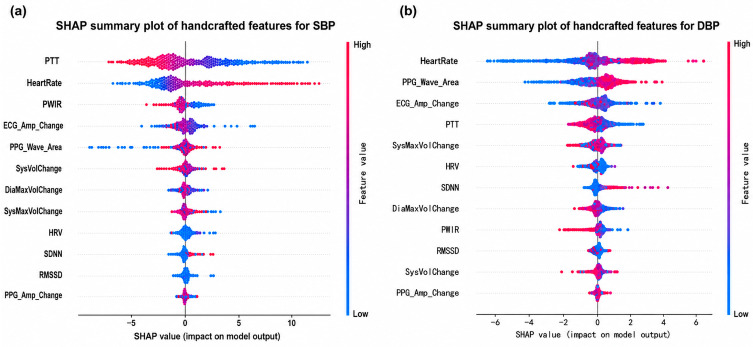
SHAP-based interpretation of handcrafted features for BP estimation. (**a**) Summary plot of feature importance for SBP prediction. (**b**) Summary plot of feature importance for DBP prediction. Features are ranked according to their overall contribution to the model output. The color of each point denotes the feature value, and the SHAP value represents the direction and magnitude of its effect on prediction. The vertical reference line denotes a SHAP value of zero; points to the right and left indicate positive and negative contributions to the model prediction, respectively.

Figure 2 shows the SHAP summary plots of candidate handcrafted features for SBP and DBP estimation. For SBP prediction, PTT and HeartRate showed the largest overall contributions, indicating the importance of pulse propagation and cardiac rhythm information. For DBP prediction, HeartRate and PPG_Wave_Area showed relatively higher importance, suggesting that rhythm-related and waveform morphology information contributed more strongly to DBP estimation. Based on SHAP ranking, physiological interpretability, cross-task consistency, and feature redundancy, PTT, HeartRate, SysVolChange, SysMaxVolChange, DiaMaxVolChange, PWIR, HRV, SDNN, and RMSSD were retained as the final handcrafted feature set. The feature selection process was performed only within the training set to avoid information leakage.

Based on SHAP ranking, physiological interpretability, cross-task consistency between SBP and DBP estimation, and feature redundancy, PTT, HeartRate, SysVolChange, SysMaxVolChange, DiaMaxVolChange, PWIR, HRV, SDNN, and RMSSD were retained as the final handcrafted feature set. The final selection was not determined solely by the absolute SHAP ranking in Figure 2, but was intended to construct a compact group-level representation covering time-delay, heart-rate, PPG morphology, and short-term rhythm-variability descriptors.

Although ECG_Amp_Change showed noticeable SHAP importance, amplitude-related information may overlap with other waveform morphology descriptors and can also be partly captured by the raw-waveform self-supervised representation. Therefore, ECG_Amp_Change was not retained as an independent handcrafted feature. RMSSD was retained to preserve short-term rhythm-variability information complementary to HeartRate and SDNN.

### 2.5. Self-Supervised Waveform Representation Module

The self-supervised waveform representation module was designed to capture temporal patterns that cannot be fully described by handcrafted features. Each valid ECG/PPG window was arranged as a two-channel input:

(3) $$X_{\mathrm{raw}}\in R^{2\times T}$$

where T denotes the number of samples in each window. Before encoding, each channel was normalized within the window to reduce amplitude variations across subjects and recording conditions.

A 1D convolutional patch embedding layer was used to divide the input sequence into non-overlapping local patches. The kernel size and stride were both set to the patch size (P = 50) samples, resulting in N = T/P patch tokens. The patch tokens were projected to a 64-dimensional embedding space, and positional encoding was added before the Transformer encoder. The encoder contained two Transformer layers with four attention heads and was used to model temporal dependencies across patches.

During self-supervised pretraining, 40% of the patch tokens were randomly masked and replaced by learnable mask tokens. A lightweight reconstruction decoder was then used to recover the masked patches from the unmasked contextual tokens. Given the masked patch set *M*, the reconstruction loss was defined as

(4) $$L_{\mathrm{ssl}}=\frac{1}{\left|M\right|}\sum_{i\in M}{\left\|{\hat{p}}_{i}-p_{i}\right\|}_{2}^{2}$$

where ${\hat{p}}_{i}$ and $p^{i}$ denote the original and reconstructed values of the i-th masked patch, respectively. The encoder was pretrained for 10 epochs using the Adam optimizer with a learning rate of 10^−4^ and a batch size of 16. After pretraining, only the encoder was retained as the waveform feature extractor, while the decoder was discarded.

**Figure 3:**
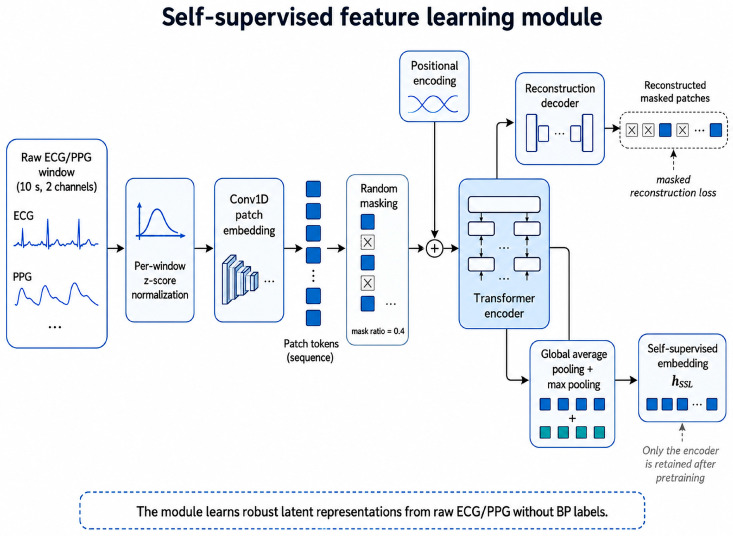
Self-supervised feature learning module framework. Ellipses indicate omitted intermediate waveform samples, patch tokens, reconstructed patches, and embedding dimensions for schematic clarity.

The encoder output was aggregated using global average pooling and global max pooling, and the two pooled vectors were concatenated as

(5) $$h_{\mathrm{SSL}}={\left[h_{\mathrm{avg}}^{T},h_{\max}^{T}\right]}^{T}\in R^{128}$$

where $h_{\mathrm{avg}}$ and $h_{\max}$ denote the global-average-pooled and global-max-pooled encoder outputs, respectively. $h_{\mathrm{SSL}}$ denotes the self-supervised waveform representation of the ECG/PPG window. To prevent information leakage, self-supervised pretraining was performed only on ECG/PPG windows from the training subjects in each cross-validation fold. The architecture of the self-supervised feature learning module is illustrated in Figure 3.

### 2.6. Personalized Calibration and Feature Fusion

The personalized calibration module was introduced to account for inter-subject differences in baseline BP. For each subject, the earliest recording file was used as the calibration file. The mean SBP and DBP values in this file were calculated as the subject-specific base BP. The calibration file was used only to estimate subject-specific prior information and calibration parameters, and was excluded from the final performance evaluation. For the BP-UCI dataset, explicit subject-specific calibration files were not available. Therefore, calibration was implemented using the initial reference BP information available within each selected record, and the calibrated segment was excluded from the evaluation set. This setting was used to approximate a practical calibration scenario rather than a fully subject-specific longitudinal calibration protocol.

The personalized calibration was performed by combining the model prediction with the subject-specific baseline BP:

(6) $$\tilde{y}=(1-\alpha )\hat{y}+\alpha b$$

where $\hat{y}$ is the uncalibrated BP prediction, b is the subject-specific baseline BP obtained from the calibration file, $\tilde{y}$ is the calibrated BP prediction, and α is the calibration weight.

The balance factor α controls the contribution of the uncalibrated prediction and the subject-specific baseline BP. A larger α gives more weight to baseline BP, whereas a smaller α relies more on the model output. The calibration file was used only to estimate baseline BP and calibration parameters and was excluded from final evaluation.

After calibration, the handcrafted features, self-supervised waveform representation, and subject-specific baseline BP information were integrated for final BP estimation. Let $h\in R^{9}$ denote the handcrafted feature vector, which contained nine selected physiological features, including PTT, HeartRate, SysVolChange, SysMaxVolChange, DiaMaxVolChange, PWIR, HRV, SDNN, and RMSSD. Let $h_{\mathrm{SSL}}\in R^{128}$ denote the self-supervised waveform representation obtained by concatenating the global average pooling and global max pooling outputs of the encoder. The subject-specific baseline BP vector was denoted as $b={\left[b_{1},b_{2}\right]}^{⊤}$, where $b_{1}$ and $b_{2}$ represent the baseline systolic and diastolic BP values obtained from the calibration file, respectively. Therefore, the final fused feature vector was defined as

(7) $$f={\left[h^{T},h_{\mathrm{SSL}}^{T},b^{T}\right]}^{T}\in R^{139}$$

where f denotes the final fused feature vector used as the input to the XGBoost regression models.

Before regression, missing feature values were imputed, and feature standardization was fitted only on the training set and then applied to the test set. Considering the limited sample size, XGBoost was adopted as the structured regression model rather than using a large end-to-end deep regression head. Two independent XGBoost regressors were trained to estimate SBP and DBP, respectively. This module outputs the final BP estimates by combining interpretable physiological features, latent waveform representations, and personalized BP information within a unified structured prediction framework.

### 2.7. Experimental Settings and Evaluation Metrics

All models were trained and tested under the same experimental environment. For the private dataset, subject-wise grouped five-fold cross-validation was adopted to ensure subject independence during evaluation. Specifically, all subjects were divided into five mutually exclusive groups. In each fold, four groups were used for training and the remaining group was used for testing. This process was repeated five times, and the final results were reported as the mean ± standard deviation across folds. Samples from the same subject were assigned exclusively to either the training set or the test set to avoid subject-level data leakage.

For personalized calibration, all records of each test subject were sorted according to acquisition time. The earliest file was used as the calibration file, and the remaining files were used for performance evaluation. The calibration file was used only to estimate subject-specific prior BP information and calibration parameters, and was excluded from the final evaluation. This protocol simulates a practical scenario in which a user first provides an initial recording with reference BP values for calibration and then undergoes continuous BP estimation on subsequent recordings.

Machine learning models were implemented using Scikit-learn, and the self-supervised representation learning module was implemented using PyTorch 2.10.0. For the structured regression module, two independent XGBoost regressors were trained for SBP and DBP estimation. The main XGBoost parameters were set as follows: the number of trees was 100, the maximum tree depth was 5, and the learning rate was 0.1. The same parameter setting was used for the baseline XGBoost and Fusion-XGB models to ensure a fair comparison. Missing values were imputed using the training-set statistics, and feature standardization was fitted only on the training set and then applied to the test set. All experiments were conducted on a workstation equipped with an NVIDIA RTX 4060 GPU.

Mean absolute error (MAE) and root mean square error (RMSE) were used as the primary evaluation metrics:

(8) $$\mathrm{MAE}=\frac{1}{N}\sum_{i=1}^{N}\left|y_{i}-{\hat{y}}_{i}\right|,$$

(9) $$\mathrm{RMSE}=\sqrt{\frac{1}{N}\sum_{i=1}^{N}{\left(y_{i}-{\hat{y}}_{i}\right)}^{2}}$$

where $y_{i}$ and ${\hat{y}}_{i}$ denote the reference and predicted BP values of the i-th test sample, respectively.

MAE and RMSE were used as empirical prediction error metrics on the held-out test folds, rather than as unbiased estimators of population-level error variance. To explicitly evaluate systematic prediction bias and error dispersion, mean error (ME) and standard deviation of error (SDE) were additionally reported together with AAMI/ISO and BHS-style evaluation criteria.

## 3. Results

### 3.1. Performance Comparison

Table 3 presents the five-fold cross-validation results on the private dataset. Overall, Fusion-XGB achieved the best balanced performance across SBP and DBP. The proposed model obtained an SBP MAE of 7.25 ± 0.86 mmHg and a DBP MAE of 3.98 ± 0.72 mmHg, outperforming the baseline XGBoost model on both targets. The improvement was more pronounced for DBP estimation, suggesting that the self-supervised waveform representation provided complementary information beyond handcrafted physiological features. Although Ridge Regression achieved a competitive SBP RMSE, Fusion-XGB showed more consistent performance across both SBP and DBP. These results indicate that combining handcrafted features, self-supervised waveform representations, and subject-specific prior BP information can improve estimation stability under limited sample size and inter-subject variability.

Table 4 shows the five-fold cross-validation results on the BP-UCI dataset. Compared with the private dataset, BP-UCI contains a more heterogeneous distribution and a wider BP range, which makes it useful for evaluating model performance under external data conditions. Fusion-XGB improved SBP-related metrics compared with the XGBoost baseline, indicating that the proposed fusion and calibration strategy maintained effective predictive capability on the external dataset. For DBP estimation, several conventional models achieved competitive or slightly better results, suggesting that the optimal modeling strategy may differ between SBP and DBP under different data distributions.

Overall, Fusion-XGB did not outperform all comparison models across every metric. Nevertheless, it consistently improved over the XGBoost baseline and maintained stable performance under BP-UCI evaluation. These findings support the potential value of combining handcrafted features, self-supervised representations, and calibration information for cuffless BP estimation, while also indicating that further validation on larger and more diverse datasets is needed.

### 3.2. Ablation Studies

To analyze the contributions of feature fusion and personalized calibration, ablation studies were conducted on both the private dataset and the BP-UCI dataset. As shown in Table 5, compared with the baseline model without feature fusion or calibration, the full model reduced SBP MAE from 9.47 to 7.25 mmHg and DBP MAE from 5.01 to 3.98 mmHg on the private dataset, corresponding to relative reductions of approximately 23.4% and 20.6%, respectively.

Introducing the self-supervised feature fusion branch reduced the average errors of both SBP and DBP, indicating that latent waveform representations learned from raw ECG/PPG signals can provide complementary information beyond handcrafted features. Personalized calibration further improved SBP estimation by compensating for inter-subject baseline BP shifts. The best overall performance was obtained when feature fusion and personalized calibration were used together, suggesting that the two components play complementary roles.

A similar trend was observed on the BP-UCI dataset, as shown in Table 6. The full model reduced SBP MAE from 9.54 to 7.85 mmHg and DBP MAE from 4.53 to 4.02 mmHg compared with the non-fusion and non-calibration baseline. These results confirm that the proposed framework benefits from the joint use of structured physiological priors, self-supervised temporal representations, and personalized BP information.

### 3.3. Clinical Evaluation and Bland–Altman Analysis

Table 7 presents the clinical evaluation results of the calibrated model on the private and BP-UCI datasets. For DBP estimation, the model satisfied the numerical AAMI/ISO error criterion and achieved BHS Grade A on both datasets. In contrast, SBP estimation did not meet the AAMI/ISO criterion, with larger SDE values and lower BHS grades. These results suggest that the proposed framework provided more stable agreement for DBP estimation than for SBP estimation.

**Figure 4:**
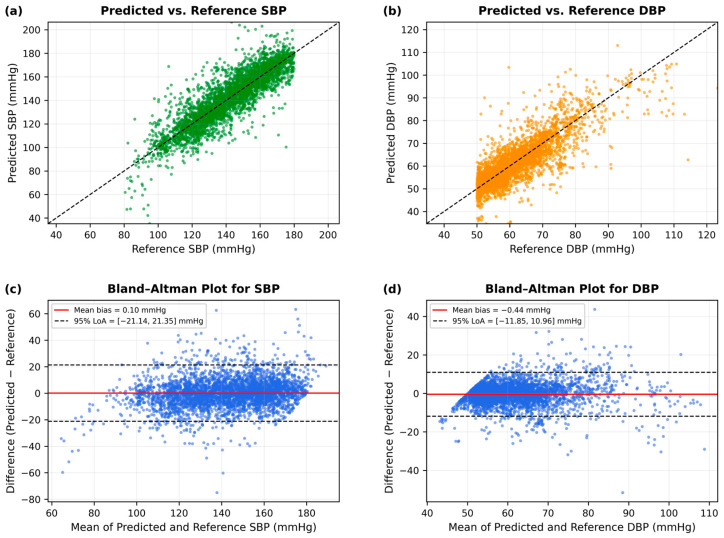
Representative prediction agreement and Bland–Altman analysis of the calibrated Fusion-XGB model on the BP-UCI dataset using the fifth cross-validation fold. (**a**) Predicted versus reference SBP. (**b**) Predicted versus reference DBP. (**c**) Bland–Altman analysis for SBP estimation. (**d**) Bland–Altman analysis for DBP estimation. The diagonal dashed lines in the scatter plots denote the identity line, while the solid and dashed horizontal lines in the Bland–Altman plots denote the mean bias and 95% limits of agreement, respectively.

To further illustrate prediction agreement, representative predicted versus reference plots and Bland–Altman analyses were performed for the calibrated Fusion-XGB model, as shown in Figure 4. The results show that DBP estimation exhibited a narrower error distribution and better agreement than SBP estimation, whereas SBP errors showed larger dispersion, consistent with the clinical evaluation results.

## 4. Discussion

The experimental results demonstrate that the proposed Fusion-XGB framework can improve cuffless BP estimation by combining handcrafted physiological features, self-supervised waveform representations, and subject-specific prior BP information. This design preserves physiologically interpretable information while using self-supervised learning to capture latent temporal patterns that are difficult to explicitly describe with handcrafted features.

Experimental results on the private dataset showed that Fusion-XGB improved BP estimation compared with baseline models, particularly for DBP estimation. This suggests that self-supervised waveform representations provide complementary information to PTT, HRV, and PPG morphological descriptors, and can improve robustness when handcrafted features are affected by signal quality or fiducial point detection. On the BP-UCI dataset, Fusion-XGB maintained stable predictive performance under an external data distribution and consistently improved over the XGBoost baseline, although it did not outperform all comparison models on every metric. Because existing studies differ substantially in dataset size, signal modality, subject split strategy, calibration protocol, and reference BP acquisition, direct numerical comparison with previous methods remains difficult.

From the clinical evaluation perspective, DBP estimation achieved BHS Grade A and satisfied the numerical AAMI/ISO error criterion, whereas SBP estimation did not fully meet the AAMI/ISO requirement and showed lower BHS grading. The larger dispersion of SBP errors may be associated with limited sample size, inter-subject variability, motion-induced waveform changes, and intermittently measured cuff BP labels. These findings indicate that the proposed method provides more stable agreement for DBP estimation, while further improvement is still needed for clinically reliable SBP monitoring.

Several limitations should be noted. Although subject-wise cross-validation was adopted to reduce subject-level data leakage, the limited cohort size and relatively narrow participant distribution may still restrict the generalizability of the findings. The private dataset was collected under controlled laboratory conditions, and the subject population did not sufficiently include elderly individuals, hypertensive patients, obese subjects, or patients with cardiovascular comorbidities. In addition, the current personalized calibration strategy relied on the earliest calibration recording and used a simple alpha-weighted correction. For BP-UCI, calibration was approximated using an initial-segment protocol because explicit longitudinal calibration files were not available. Future work will focus on adaptive calibration strategies and the integration of motion-related signals to improve robustness during daily activities.

## 5. Conclusions

This study proposed a personalized calibration and hybrid feature fusion framework for continuous BP estimation using ECG and PPG signals. By integrating handcrafted physiological features, self-supervised waveform representations, and subject-specific prior BP information, the proposed Fusion-XGB model improved estimation performance over baseline models on both the private dataset and the BP-UCI dataset. The results suggest the feasibility and potential of the proposed framework for small-sample BP estimation, particularly for DBP prediction. However, further validation on larger, more diverse, and real-world datasets is still required before clinical deployment, especially for improving SBP accuracy, calibration robustness, and cross-population generalization.

## Figures and Tables

**Table 1:** Demographic characteristics of participants in the private dataset.

Characteristic	Value
Number of subjects	45
Sex	33 males/12 females
Age (years)	23.7 ± 4.0 (range 19–28)
Height (cm)	172.4 ± 7.2 (range 156–183)
Weight (kg)	68.9 ± 10.7 (range 44–87)
BMI (kg/m^2^)	23.0 ± 2.4 (range 17.2–27.8)
SBP (mmHg)	125.7 ± 14.5 (87–189)
DBP (mmHg)	74.0 ± 6.7 (48–104)
Recording condition	Rest and post-exercise recovery
Sampling frequency	1000 Hz

**Table 2:** Handcrafted feature groups used in this study.

Feature Group	Features	Description
Time-delay	PTT	Time interval between ECG R peak and PPG systolic peak
Heart rate	HeartRate	Average heart rate derived from RR intervals
PPG morphology	SysVolChange, SysMaxVolChange, DiaMaxVolChange, PWIR	Pulse amplitude and slope-related waveform descriptors
HRV	HRV, SDNN, RMSSD	Short-term RR interval variability descriptors

**Table 3:** Performance comparison of different models on the private dataset (mean ± standard deviation over five folds).

Model	SBP MAE	SBP RMSE	DBP MAE	DBP RMSE
Ridge Regression	7.37 ± 1.08	**9.14 ± 1.24**	4.76 ± 0.96	6.23 ± 1.17
Decision Tree	7.76 ± 0.74	9.70 ± 1.08	5.64 ± 1.60	7.19 ± 1.79
SVR	7.61 ± 1.13	9.55 ± 1.54	4.96 ± 1.23	6.44 ± 1.35
Random Forest	7.34 ± 0.95	9.78 ± 0.89	5.31 ± 1.27	6.90 ± 1.42
XGBoost	7.30 ± 1.22	9.75 ± 1.23	5.31 ± 1.07	6.87 ± 1.28
Fusion-XGB	**7.25 ± 0.86**	9.26 ± 1.02	**3.98 ± 0.72**	**5.18 ± 0.88**

Note: Throughout all tables, bold values indicate the best performance for each evaluation metric.

**Table 4:** Performance comparison of different models on the BP-UCI dataset under bias calibration (mean ± standard deviation over five folds).

Model	SBP MAE	SBP RMSE	DBP MAE	DBP RMSE
Fusion-XGB	**7.85 ± 0.33**	**11.02 ± 0.56**	4.02 ± 0.13	6.15 ± 0.39
XGBoost (baseline)	8.32 ± 0.22	11.77 ± 0.43	4.05 ± 0.07	6.20 ± 0.24
Ridge Regression	8.22 ± 0.19	11.67 ± 0.39	4.19 ± 0.07	6.37 ± 0.13
SVR	8.25 ± 0.15	11.93 ± 0.32	3.88 ± 0.07	**6.03 ± 0.20**
Decision Tree	9.36 ± 0.15	13.86 ± 0.33	4.55 ± 0.08	7.34 ± 0.47
Random Forest	8.05 ± 0.27	11.74 ± 0.51	**3.83 ± 0.10**	6.14 ± 0.19

**Table 5:** Ablation study of feature fusion and personalized calibration on the private dataset.

Fusion	Calibration	SBP MAE	SBP RMSE	DBP MAE	DBP RMSE
No	No	9.47 ± 1.34	11.76 ± 1.25	5.01 ± 1.27	6.53 ± 1.42
Yes	No	8.44 ± 0.95	10.60 ± 0.84	4.89 ± 0.92	6.34 ± 1.03
No	Yes	7.30 ± 1.22	9.75 ± 1.23	5.31 ± 1.07	6.87 ± 1.28
Yes	Yes	**7.25 ± 0.86**	**9.26 ± 1.02**	**3.98 ± 0.72**	**5.18 ± 0.88**

**Table 6:** Ablation study of feature fusion and personalized calibration on the BP-UCI dataset.

Fusion	Calibration	SBP MAE	SBP RMSE	DBP MAE	DBP RMSE
No	No	9.54 ± 0.19	12.76 ± 0.24	4.53 ± 0.10	6.73 ± 0.17
Yes	No	8.87 ± 0.30	11.84 ± 0.30	4.26 ± 0.19	6.26 ± 0.34
No	Yes	8.32 ± 0.22	11.77 ± 0.43	4.05 ± 0.07	6.20 ± 0.24
Yes	Yes	**7.85 ± 0.33**	**11.02 ± 0.56**	**4.02 ± 0.13**	**6.15 ± 0.39**

**Table 7:** Clinical evaluation of the calibrated model on the private and BP-UCI datasets.

Dataset	BP	ME (mmHg)	SDE (mmHg)	AAMI/ISO	CPE5 (%)	CPE10 (%)	CPE15 (%)	BHS
Private	SBP	−0.98	8.89	Not met	51.8	78.9	90.2	B
	DBP	1.55	5.31	Met	62.0	94.2	99.3	A
BP-UCI	SBP	−0.23	10.73	Not met	45.6	73.6	87.4	C
	DBP	−0.40	5.76	Met	73.2	91.9	97.7	A

Note: ME, mean error; SDE, standard deviation of error; CPE5/10/15, cumulative percentage of absolute errors within 5/10/15 mmHg.

## Data Availability

The public BP-UCI dataset used in this study is available from the UCI Machine Learning Repository: https://doi.org/10.24432/C5B602. It is also accessible from Kaggle: https://www.kaggle.com/datasets/mkachuee/BloodPressureDataset, accessed on 1 June 2026. The private dataset generated and analyzed during the current study is not publicly available due to participant privacy and ethical restrictions, but may be available from the corresponding author upon reasonable request.
